# MRI for collateral assessment pre-thrombectomy and association with outcome: a systematic review and meta-analysis

**DOI:** 10.1007/s00234-023-03127-8

**Published:** 2023-02-27

**Authors:** Sarah Emhemed Abousrafa, Grant Mair

**Affiliations:** 1 College of Medicine and Veterinary Medicine, University of Edinburgh, Edinburgh, UK; 2 Centre for Clinical Brain Sciences, Chancellor’s Building, University of Edinburgh, 49 Little France Crescent, Edinburgh, EH16 4SB UK

**Keywords:** Magnetic resonance imaging, Stroke, Collateral status, Endovascular thrombectomy

## Abstract

**Purpose:**

Various neuroimaging methods exist to assess the collateral circulation in stroke patients but much of the evidence is based on computed tomography. Our aim was to review the evidence for using magnetic resonance imaging for collateral status evaluation pre-thrombectomy and assess the impact of these methods on functional independence.

**Methods:**

We systematically reviewed EMBASE and MEDLINE for studies that evaluated baseline collaterals using MRI pre-thrombectomy and conducted a meta-analysis to express the relationship between good collaterals (defined variably as the presence [good] vs absence [poor] or quality [ordinal scores binarized as good-moderate vs poor] of collaterals) and functional independence (modified Rankin score mRS≤2) at 90 days. Outcome data were presented as relative risk (RR, 95% confidence interval, 95%CI). We assessed for study heterogeneity, publication bias, and conducted subgroup analyses of different MRI methods and affected arterial territories.

**Results:**

From 497 studies identified, we included 24 (1957 patients) for the qualitative synthesis, and 6 (479 patients) for the metanalysis. Good pre-thrombectomy collaterals were significantly associated with favorable outcome at 90 days (RR=1.91, 95%CI=1.36–2.68], *p*= 0.0002) with no difference between MRI methods and affected arterial territory subgroups. There was no evidence of statistical heterogeneity (*I*^2^=25%) among studies but there was evidence of publication bias.

**Conclusion:**

In stroke patients treated with thrombectomy, good pre-treatment collaterals assessed using MRI are associated with double the rate of functional independence. However, we found evidence that relevant MR methods are heterogenous and under-reported. Greater standardization and clinical validation of MRI for collateral evaluation pre-thrombectomy are required.

**Supplementary Information:**

The online version contains supplementary material available at 10.1007/s00234-023-03127-8.

## Introduction

For ischemic stroke patients with large vessel occlusion, urgent treatment with endovascular thrombectomy (EVT) is associated with higher rates of recovery and return of functional independence compared to best medical care alone [1]. Individual patient-level meta-analysis of imaging data from seven randomized controlled trials (RCTs) showed that the favorable outcome effect of EVT was more likely observed in patients with better collaterals [2]. In addition, neuroimaging assessment of the arterial collateral circulation may be used to extend stroke onset to treatment time [3].

While previous systematic reviews demonstrate the importance of pre-treatment collateral status (CS) on outcome, only two included magnetic resonance imaging (MRI) methods but neither correlated with outcome [4, 5]. Those that did find associations between CS and outcome were computed tomography (CT) and digital subtraction angiography (DSA) based [6, 7], and one only included patients treated with thrombolysis [8]. Since seven out of eleven thrombectomy trials utilized MRI [9–15], a thorough review focusing on the role of MRI is warranted.

On MRI, direct angiographic methods provide structural assessment of collateral vasculature/flow using magnetic resonance angiography (MRA) sequences while indirect methods assess blood perfusion in the affected region (area of hypoperfusion) as a surrogate for collaterals. It is not clear which of these approaches is most clinically relevant. While various collateral grading systems have been proposed for CT and DSA to define what constitutes good versus poor collaterals, there is no consensus for collateral scoring using MRI [16].

We aimed to systematically review the literature where MRI was used to evaluate CS pre-thrombectomy in patients with ischemic stroke, to provide a comprehensive qualitative description of the available MRI methods, and to define what constitutes a good collateral circulation on MRI, and a meta-analysis seeking associations between CS MRI and functional independence 90 days after thrombectomy.

## Materials and methods

This systematic review was carried out and is presented according to the Preferred Reporting Items for Systematic Reviews and Meta-Analyses 2020 (PRISMA), Supplement Table 1 [17].

### Eligibility criteria

A study was deemed eligible for inclusion based on the following criteria: (a) ischemic stroke patients with large vessel occlusion (LVO); (b) observational cohorts and post hoc analyses of RCTs; (c) evaluations of CS using MRI; (d) patients treated with thrombectomy (with or without tissue plasminogen activator (tPA)); (e) MRI methodology sufficiently described; (f) prospective or retrospective. Additionally, for quantitative meta-analysis, we included studies if (a) the number of cases with good versus poor collaterals could be extracted and (b) associations between good/poor collaterals and functional independence at 90 days were reported. The following study types were excluded: (a) case reports, conference abstracts/papers, systematic reviews, letters, comments, and animal studies; (b) where CS was not assessed by MRI; (c) if patients were not treated with thrombectomy; (d) where other arterial abnormalities were the focus, e.g., moyamoya disease or carotid stenosis; (e) if total thrombectomy treated patients were <10; (f) technical reports on healthy volunteers. Studies were screened carefully for publications with similar cohorts, e.g., post hoc analyses of the same RCTs.

### Search strategy

We searched MEDLINE and EMBASE for full-text articles in English from inception till 31st of October 2022. Also, references of relevant studies and reviews were searched for additional potential publications. In brief, the search strategy is composed of five distinct terms: (1) stroke, (2) thrombectomy, (3) collaterals, (4) MRI, (5) statistical terms linked using the AND Boolean operator. Additionally, limits were applied to filter studies according to design. The full search strategy (with limits) can be viewed in supplemental table 2.

### Study selection process

Titles and abstracts from both databases were screened successively for minimum eligibility. One researcher (SEA) independently screened records and extracted relevant data. First, duplicates were automatically removed from the total of all publications identified using ENDNOTE 20 (Clarivate, Philadelphia, USA). Lastly, the titles and abstracts of the remaining studies were thoroughly assessed, and suitable papers were selected for full-text evaluation. To avoid over exclusions, any study using MRI to assess the CS was considered the minimum requirement for full-text review.

### Data extraction process and quality assessment

Data were extracted manually employing a standardized extraction form, supplemental table 3. For all studies selected for full-text review, we collected (a) author and year of publication; (b) number of patients treated with thrombectomy; (c) study design; (d) cut-off used to dichotomize CS as good vs poor; (e) collateral grading system; (f) magnetic field strength; (g) the number of patients with good and poor collaterals, however defined by authors or where this could be derived—in other words, we accepted all published definitions of collateral quality; (h) stroke onset time; and to allow for sensitivity analyses, (a) number of patients who achieved functional independence at 90 days for each CS group; (b) affected arterial territory; (c) MRI method. When more than one treatment was included, only data for thrombectomy patients were extracted. Also, when more than one imaging modality was used, only data on MRI was extracted. For meta-analysis, when CS was reported in more than 2 categories, these were dichotomized into good and poor (defined variably as the presence and/or quality of collaterals). The detailed dichotomization process for each study is included in supplemental table 4. Quality was assessed using the Newcastle-Ottawa scale for non-randomized trials (NOS) where studies with a score of 6 or higher are considered of high quality [18]. Publication bias was evaluated by visual inspection of a funnel plot.

### Statistical analysis and data synthesis

Data were analyzed using the Cochrane Review Manager (RevMan) version 5.4. The association between good/poor pre-treatment collaterals and functional independence is displayed using a random effects model and 95% confidence interval (95%CI) with risk ratio (RR) as the effect measure. Study heterogeneity was tested using *I*^2^ where >50% is considered substantial [19]. Sensitivity analyses were performed for (a) the arterial territory affected and (b) the MR method used. Data are visually presented using a forest plot.

## Results

### Search strategy

The search yielded a total of 497 studies (311 MEDLINE and 186 EMBASE), while searching references of review articles provided 5 further studies. Forty-four studies were removed for duplication and 218 for irrelevant publication types. The remaining 240 studies were title and abstract screened resulting in 47 studies undergoing full-text assessment and where appropriate, data extraction.

### Study selection

**Fig. 1:**
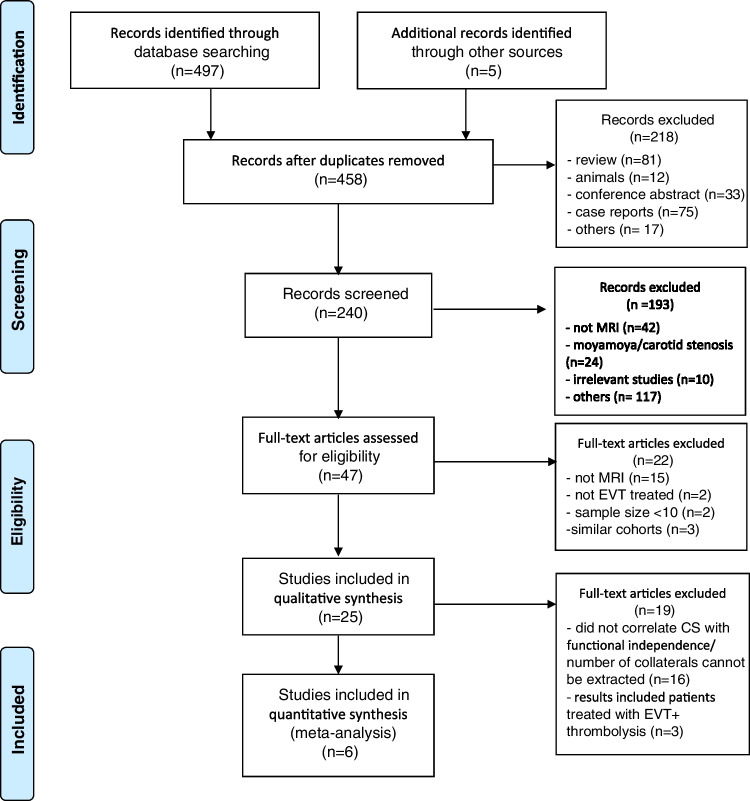
PRISMA flow diagram

Following full-text review, two publications were noted to have substantial similarities in cohorts, methods, and image analysis techniques [20, 21], two studies pooled data from ASTER and THRACE [22, 23], and two studies pooled data from DEFUSE3 [24, 25]. Only one from each pair of overlapping studies was included [20, 23, 24]. Finally, 25 unique studies were eligible for the qualitative synthesis, and 6 for the meta-analysis. The PRISMA 2009 flow diagram can be seen in Fig. 1

### Study characteristics

The general characteristics of the selected studies can be seen in Table 1. Included studies were published between the years 2013 and 2022. Two studies were prospective in design [26, 27], and 23 were retrospective [20, 23, 24, 28–47]. We included a total of 2467 participants treated with thrombectomy. Thirteen studies (432 participants) recorded the number of patients that had tPA administered along with EVT [26, 28, 33, 35, 36, 38–40, 42–46]. Enrolled participants had a stroke onset time that ranged between 4.5 and 6 h in 4 studies [20, 26, 39, 41], between 6 and 16 h in 6 studies [24, 35, 37, 38, 44, 46], and up to 24 h in 1 study [32]. Studies enrolled participants with the following arterial territory involvement (a) anterior circulation stroke in 23 studies [20,23,24,26,27,29–33,35–44,45–47); (b) posterior circulation stroke in 1 study [28]; (c) LVO not otherwise specified in 1 study [34]. Using the Newcastle-Ottawa scale for quality assessment of the 6 studies for meta-analysis, 5 studies were considered high quality with a score of 6 and higher and 1 study with medium quality with a score of 5. Results and breakdown of these 6 studies can be seen in supplemental table 5.

**Table 1:** General characteristics

Author	Year	Number of EVT treated patients	EVT + tPA	Study design	Onset time (hours)	Arterial territory
Tsui B at al. [43]	2022	104	13	Retrospective	-	Anterior circulation PAO
Faizy et al. [47]	2021	510	-	Retrospective	-	LVO of ICA or M1 or M2
Maruyama et al. [45]	2021	35	14	Retrospective	-	M1 occlusion
Derraz I et al. [44]	2021	302	162	Retrospective	8	Anterior circulation LVO
Kim HJ et al. [46]	2020	89	49	Retrospective	8	Anterior circulation
Rao V et al. [24]	2020	62	-	Retrospective	6-16	MCA or ICA
Mahmoudi M et al. [28]	2020	110*	41	Retrospective	-	Acute BAO
Guenego A et al. [29]	2020	52	-	Retrospective	-	M1 MCA
Shin J et al. [26]	2020	52	23	Prospective	6	ICA or MCA
Eker O et al. [30]	2019	240	-	Retrospective	-	Anterior circulation
Federau C et al. [31]	2019	14	-	Retrospective	-	MCA LVO
Yu I et al. [32]	2019	65	-	Retrospective	24	Anterior territory LVO
Morinaga Y et al. [33]	2019	73	40	Retrospective	-	Anterior circulation
Boujan T et al. [34]	2018	123	-	Retrospective	-	LVO
Legrand L et al. [23]	2019	100	-	Retrospective	-	Proximal MCA
Kim BJ et al. [35]	2018	60	8	Retrospective	6-12	Anterior circulation or MCA
Mahdjoub E et al. [36]	2017	36	13	Retrospective	-	MCA stroke
Nave A et al. [37]	2018	104	-	Retrospective	12	M1 MCA
Nael K et al. [38]	2018	39	18	Retrospective	9	Anterior circulation PVO
Jiang L et al. [39]	2017	55	35	Retrospective	6	ICA or MCA
Potreck A et al. [40]	2016	47	35	Retrospective	-	Solitary M1
Lou X et al. [27]	2016	19	-	Prospective	-	Acute MCA
Hernandez-Perez M et al. [41]	2016	25	-	Retrospective	>4.5	Anterior circulation LVO
Kim SJ et al. [20]	2014	94	-	Retrospective	6	Acute MCA
Nicoli F et al. [42]	2013	57	21	Retrospective	-	MCA-M1

*EVT*, endovascular thrombectomy; *tPA*, tissue plasminogen activator; *PAO*, proximal artery occlusion; *LVO*, large vessel occlusion; *ICA*, internal carotid artery; *MCA*, middle cerebral artery; *BAO*, basilar artery occlusion; *PVO*, proximal vessel occlusion

*Endovascular treatment was aborted for failed proximal/distal access in 10/110 patients

### MRI methods

**Fig. 2:**
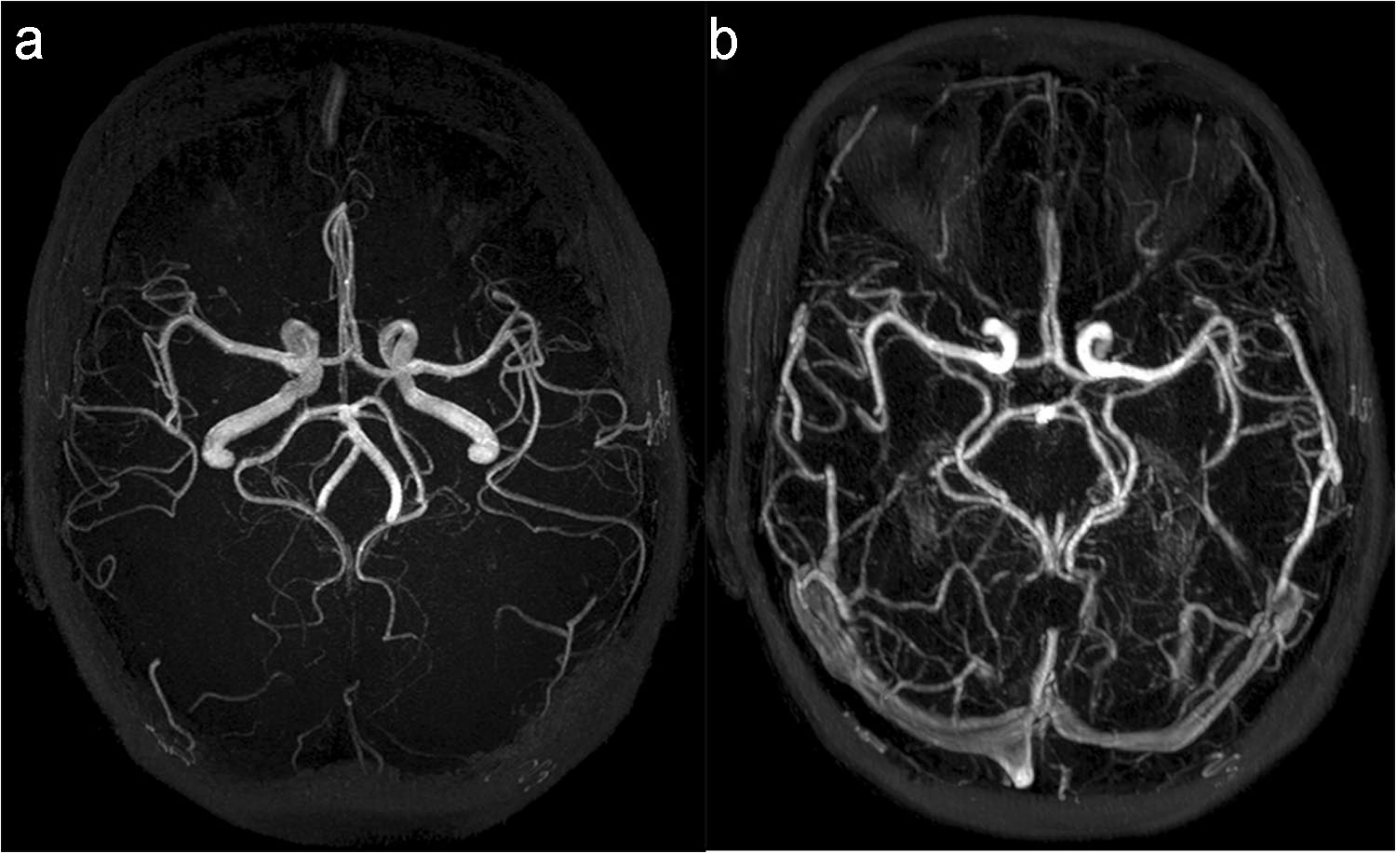
MR angiography types. **a** Time-of-flight MR angiography (TOF-MRA) and **b** contrast-enhanced MR angiography (CE-MRA). Note that for TOF-MRA since only arterial phase blood is energized prior to entering the imaging field of view, there is no venous contamination in **a**. Depending on the timing of CE-MRA, veins may be clearly visible as in **b**. This has implications for imaging collaterals since collateral flow is usually delayed

**Fig. 3:**
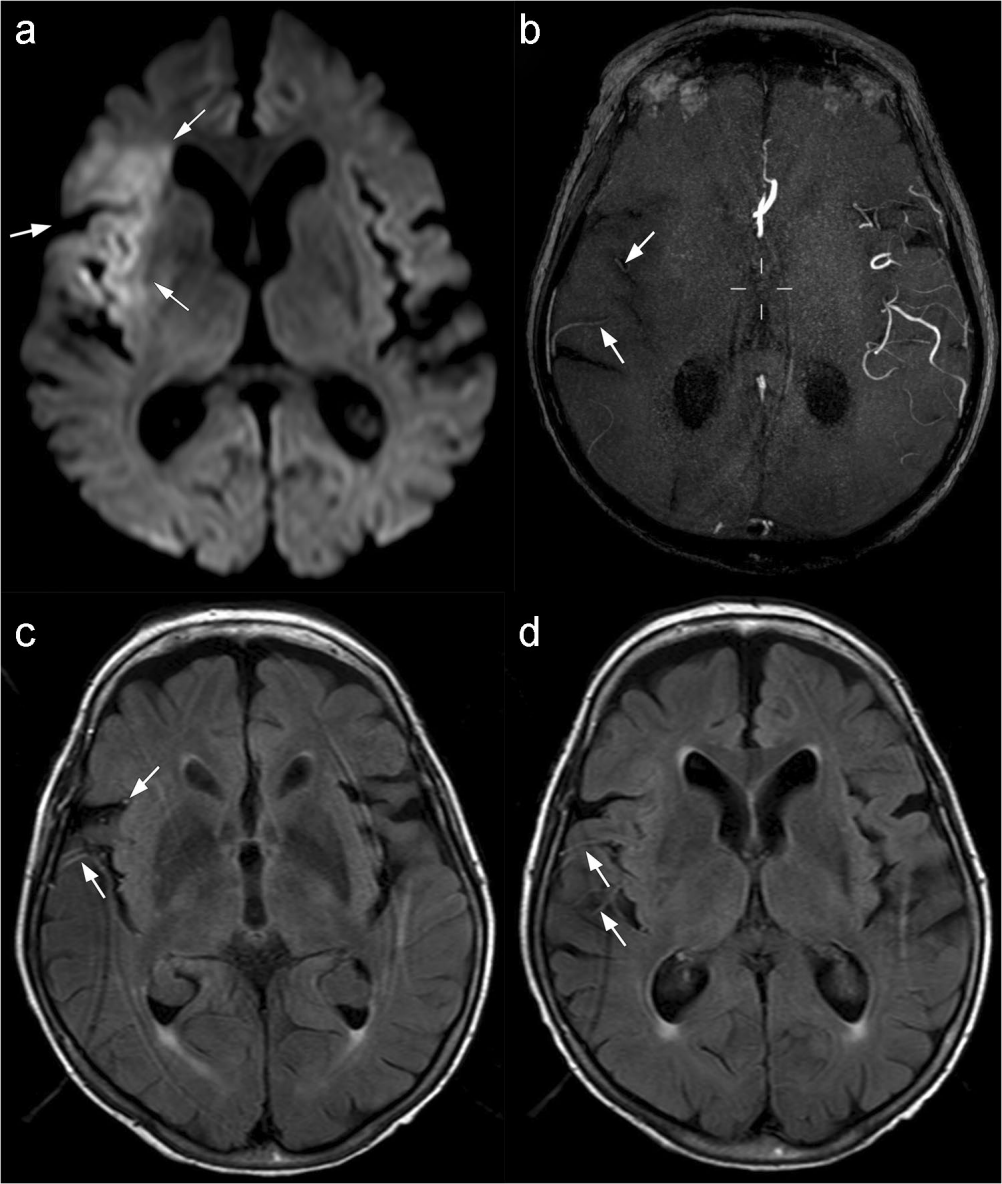
FLAIR hyperintense vessels as a means to assess collateral status. Arrows indicate **a** abnormal restricted diffusion within an acute ischemic lesion; **b** collateral vessels visible on time-of-flight MRA, note the paucity of normal middle cerebral artery branches compared with the other side of brain; **c** and **d** hyperintense vessels on FLAIR corresponding with MRA collaterals, note also the mismatch between the visibility of the ischaemic brain lesion here compared with image **a**—this diffusion-FLAIR mismatch is thought to be an indicator of ischemic tissue viability

Direct angiographic methods were employed by 7 studies [28, 33, 34, 39, 41, 43, 46], including dynamic magnetic resonance angiography (dMRA), contrast-enhanced MRA (CE-MRA), and time-of-flight MRA (TOF-MRA) (Fig. 2). Indirect methods included (a) quantification of perfusion derived collateral scores in 12 studies [20, 24, 26, 27, 29, 31, 32, 35, 38, 40, 42, 47], (b) association of CS with FLAIR hyperintense vessels (FHVs) in 5 studies (Fig. 3) [23, 36, 37, 44, 45], and (c) effect of small vessel disease (SVD) burden on pial collaterality in 1 study [30].

**Fig. 4:**
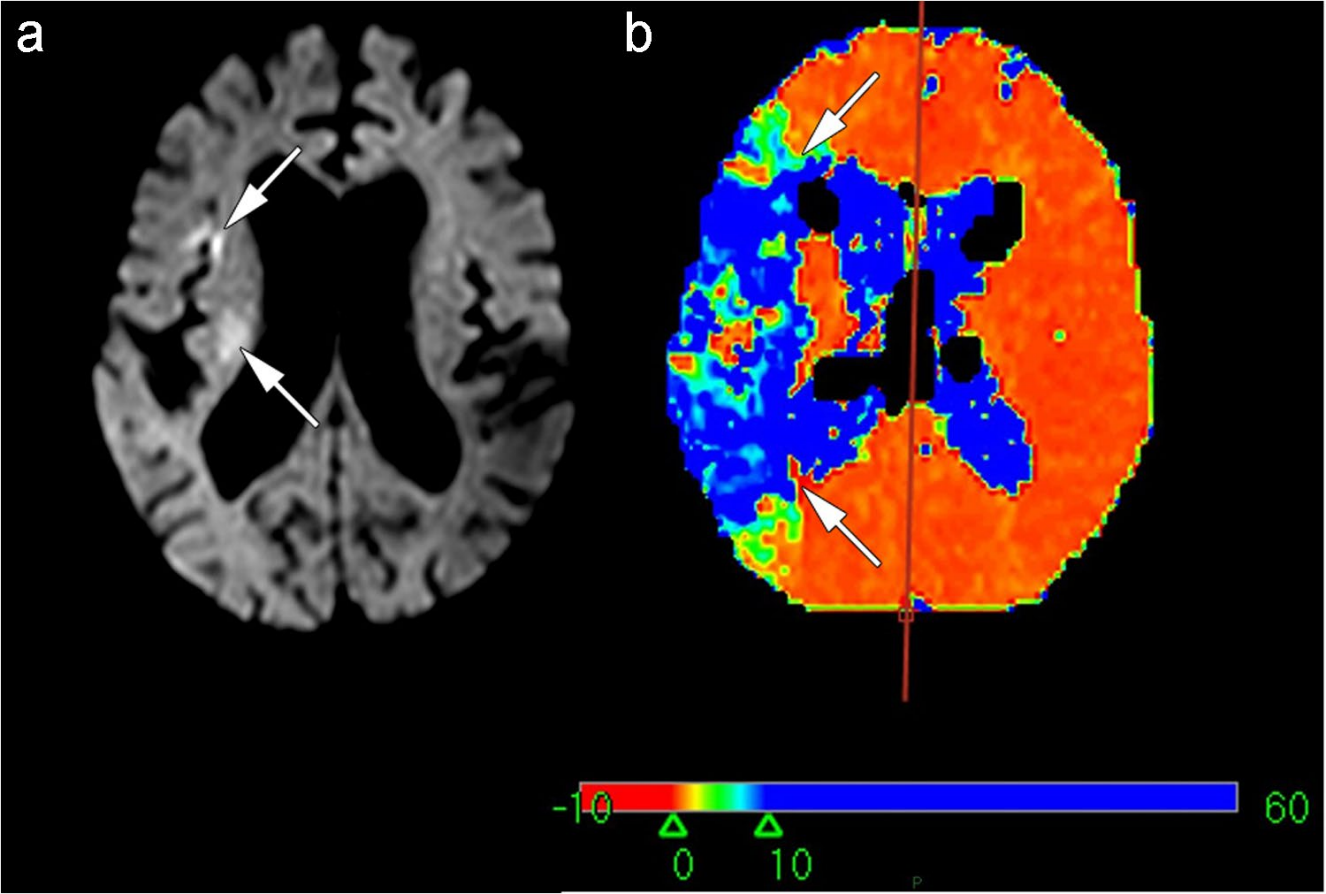
Diffusion-perfusion mismatch indicating viable ischemic brain tissue and thus indirect evidence of collateral supply. Arrows indicate **a** a small ischemic “core” lesion on diffusion-weighted imaging that is thought to be an irreversible injury and **b** a large perfusion abnormality. The difference between the ischemic lesions in **a** and **b** is thought to represent “penumbra” or reversible injury. The perfusion map in **b** is TMAX, a measure of the delay (in seconds) of contrast reaching tissue

In 7 studies, a cut-off for collateral scoring was introduced and measured using (a) receiver-operating characteristic curve (ROC) analysis of the hypoperfusion intensity ratio (HIR) resulting in an optimal threshold of HIR <0.4 as a predictor of good angiographic collaterals [29]; (b) median HIR to dichotomize CS, with HIR ≤0.35 indicating good collaterals [36]; (c) median FLAIR Hyperintense Vessel Alberta Stroke Program Early CT Score (FHV-ASPECTS) to dichotomize CS with a low FHV-ASPECTS of ≤2 indicating good collaterals [37]; (d) cSVD score ≥1 indicating severe SVD burden [30]; (e) presence of persistent salvageable tissue with a diffusion-perfusion mismatch ratio of ≥1.8 indicating favorable collaterals (Fig. 4) [35]; (f) ROC analysis of the volume of tissue with severely prolonged arterial tissue delay (VolATD6) and DWI lesion where the combination (ATD<27+DWI>15) provides the best optimal threshold of 27ml and 15 ml respectively, indicating very good angiographic collaterals [42]; (g) tissue level collaterals (TLC) measured by HIR to dichotomize CS into TLC+ (HIR≤0.4) and TLC− (HIR>0.4) [47].

A total of 14 different collateral scoring systems were used, with the most common being the American Society of Interventional and Therapeutic Neuroradiology/Society of Interventional Radiology scale (ASITN/SIR) [20, 32, 41, 42]. A full description of the MR methods used, grading systems and cut-offs can be seen in Table 2.

**Table 2:** MRI methods, grading systems, and values of good versus poor CS in relation to favorable outcome

Author	MRI method	MRI cut-off	Collateral grade	Field strength	Collateral status Good collaterals (n.) Poor collaterals (n.)	Favorable outcome (mRS) at 90 days (n.)
Rao V et al. [24]	CCS robustness calculated by HIR ratio and CBV index	**-**	**-**	3T	**-**	**-**
Mahmoudi M et al. [28]	Measuring the presence/absence of flow in the PCOMA by MRA	**-**	PCCS (0–2) 0=no PCOM 1=unilateral 2=bilateral	**-**	Good collaterals (62)	mRS (0–2)
						(25)
					Poor collaterals (48)	(10)
Guenego A et al. [29]	HIR (measured on MRP) as a surrogate marker of good DSA collaterals	Optimal threshold HIR = 0.40	Good CS HIR < 0.4 Poor CS HIR >0.4	**-**	Good collaterals (16)	**-**
					Poor collaterals (36)	
Eker O et al. [30]	SVD burden as a surrogate marker of pial collaterality approximating DSA.	(1) cSVD ≥1 severe SVD burden		1.5 or 3T	Good collaterals (136)	**-**
					Poor collaterals (104)	
Federau C et al. [31]	IVIM perfusion imaging using 6 b value DWI	Good CS Absent IVIM lesion Poor CS Present IVIM lesion	Presence or absence of IVIM penumbra	3T	Good collaterals (11)	**-**
					Poor collaterals (3)	
Yu I et al. [32]	(MRP-derived collateral map) Collateral maps are generated from source data derived from DSCE-MRP.	**-**	ASITN/SIR Poor (grade 0–1) Intermediate (grade 2) Good (grade 3–4)	3T	Good collaterals (56)	**-**
					Poor collaterals (9)	
Nicoli F et al. [42]	PWI derived collateral indices (VolATD6<27ml and DWI > 15 ml) as a surrogate marker	ATD27ml/DWI15ml Good CS ATD<27 DWI>15 Poor CS ATD>27 DWI<15	ASITN/SIR	1.5 or 3T	**-**	**-**
Morinaga Y et al. [33]	TOF-MRA to evaluate the presence or absence of ACOMA	**-**	Present vs absent ACOMA			mRS (0–3)*
					Good collaterals (52)	(25)
					Poor collaterals (21)	(3)
Boujan T et al. [34]	Employed angiographic methods (CE-MRA and 3D TOF-MRA) to visualize retrograde vessel filling of pial collaterals	**-**	3 point scale by Tan et. al. (0) none (1) poor (2) moderate/good	3T	**-**	**-**
Legrand L et al. [23]	FVH-DWI mismatch as a surrogate marker. FVH in the subarachnoid space (relative to CSF)+DWI volume.	**-**	Presence or absence of FVH-DWI mismatch	1.5 or 3T		mRS (0–2)
					Good collaterals (79)	(45)
					Poor collaterals (21)	(10)
Kim BJ et al. [35]	DWI and hypo-perfused area mismatch. volume of diffusion-restricted lesions (ADC value ≤450) and mapping of the hypo-perfused area (Tmax delay ≥6s)	(1.8) Good CS Mismatch ratio ≥1.8	**-**	**-**		mRS (0–2)
					Good collaterals (46)	(24)
					Poor collaterals (14)	
Mahdjoub E et al. [36]	Assess the extent of FHV assessed by HIR as a surrogate marker of collateral status	(0.35) Good CS HIR ≤ 0.35 Poor CS HIR > 0.35	Good CS Low HIR Poor CS High HIR	1.5T	**-**	**-**
Nael K et al. [38]	Multiparametric MRI approximating DSA PCI=volume of ATD^2–6 sec^ X rCBV^2–6 sec^	**-**	**-**	**-**	Good collaterals (22)	**-**
					Poor collaterals (-)	
Nave A et al. [37]	FHV as a surrogate marker by quantifying hyperintensities on two consecutive transverse slices.	(2) Good CS FHV-ASPECTS ≤2 Poor CS FHV-ASPECTS >2	FHV-ASPECTS (0) Seen in all territories (7) No FHV seen	1.5 or 3T	**-**	mRS (0–2)
						**-**
Jiang L et al. [39]	CE-MRA to assess collaterals sufficiency in the leptomeningeal convexity	-	5-point scale 1 Absent 5 Exuberant	3T		mRS (0–2)
					Good collaterals (10)	(7)
					Poor collaterals (45)	(11)
Potreck A et al. [40]	Computation of Tmax maps from DSC-PWI and pial volumes at delay.	-	(TMACS) Good (grade 4) Moderate (grade 3) Poor (grade 1–2)	3T		mRS (0–2)
					Good collaterals (37)	(14)
					Poor collaterals (10)	(1)
Lou X et al. [27]	ASL perfusion imaging Multi-delay 3D pCASL protocol.	-	3-point scale in 10 anatomical regions On CBV, CBF, ATT	1.5 or 3T	-	mRS=(0–3)
						(23)
Hernandez-Perez M et al. [41]	dMRA to visualize retrograde collateral filling at different delay times	-	ASITN/SIR Complete (3–4) Incomplete (0–2)	3T	Good collaterals (12)	-
					Poor collaterals (13)	
Kim S et al. [20]	Generate collateral maps based on source data derived from DSC- PWI	-	Flow map collateral grade based on ASITN/SIR	3T		mRS=(0–2)
					Good collaterals (73)	(43)
					Poor collaterals (21)	(7)
Shin J et al. [26]	PWI parameters (TTP and Tmax maps) Calculate TTP delay hypo-perfused volumes	-	-	3T	-	mRS (0–2)
						(27)
Derraz I et al. [44]	WMH burden (periventricular, deep, and total) on FLAIR in association with collateral development on DSA	-	-	1.5 or 3T	-	-
Maruyama D et al. [45]	Assess four FVH-DWI lesion patterns to stratify regional collateral flow on DSA	-		1.5T	-	-
Tsui B et al. [43]	CE-MRA and TOF-MRA	-	Maas scoring (1–2) insufficient (3–5) sufficient Tan scoring (0–1) insufficient (2–3) sufficient		Maas scoring Sufficient collaterals (2) Poor collaterals (51) Tan scoring Sufficient collaterals (22) Poor collaterals (35)	-
Kim HJ et al. [46]	By using source data from DCE-MRA, MRA collateral map was reconstructed to evaluate collateral perfusion status.	-	(MAC) scores Excellent (5) Good (4) Intermediate to good (3) Intermediate to poor (2) Poor (1) Very poor (grade 0)	3T	-	mRS (0–2)
Faizy T et al. [47]	Tissue level collaterals determined by the HIR, defined as the volume of ischemic brain tissue with a (Tmax) delay of >10seconds divided by the volume of brain tissue with a Tmax delay of >6seconds	HIR (0.4) TLC+ HIR≤0.4 TLC- HIR>0.4	Favorable collaterals HIR≤0.4 Poor collaterals HIR >0.4	-	Good collaterals (276)	-
					Poor collaterals (304)	

*mRS at discharge

*mRS*, modified Rankin score; *CCS*, collateral circulation status; *HIR*, hypoperfusion intensity ratio; *CBV*, cerebral blood volume; *PCOMA*, posterior communicating artery; *MRA*, magnetic resonance angiography; *PCCS*, posterior circulation collateral score; *MRP*, magnetic resonance perfusion; *DSA*, digital subtraction angiograph; *CS*, collateral score; *SVD*, small vessel disease; *cSVD*, cerebral SVD; *IVIM*, intravoxel incoherent motion; *DWI*, diffusion-weighted imaging; *DSCE-MRP*, dynamic susceptibility contrast-enhanced MRP; *ASITN/SIR*, American Society of Interventional and Therapeutic Neuroradiology/Society of Interventional Radiology scale; *PWI*, perfusion-weighted imaging; *VolATD*, volume arterial tissue delay; *TOF-MRA*, Time-Of-Flight magnetic resonance angiography; *ACOMA*, anterior communicating artery; *CE-MRA*, contrast-enhanced magnetic resonance angiography; *FVH-DWI*, FLAIR vascular hyperintensity-diffusion-weighted imaging; *CSF*, cerebrospinal fluid; *ADC*, attenuation deficiency coefficient; *Tmax*, time-to-maximum; *CS*, collateral status; *FHV*, FLAIR hyperintense vessel; *PCI*, perfusion collateral index; *ATD*, arterial tissue delay; *rCBV*, relative cerebral blood volume; *FHV-ASPECTS*, FLAIR hyperintense vessel-Alberta stroke programme early CT score; *CE-MRA*, contrast-enhanced MRA; *DSC-PWI*, dynamic susceptibility contrast-perfusion-weighted imaging, *TMACS*, Tmax map-assessed collateral score; *ASL*, arterial sin labelling; *pCASL*, pseudo-continuous arterial spin-labelling; *CBF*, cerebral blood flow; *ATT*, arterial transit time; *dMRA*, dynamic magnetic resonance angiography; *TTP*, time to peak; *WMH*, white matter hyperintensity; *FLAIR*, fluid-attenuated inversion recovery; *DCE-MRA*, dynamic contrast-enhanced magnetic resonance angiography; *MAC*, MR acute ischemic stroke collateral scores; *TLC*, tissue-level collaterals

### Collateral status and outcome

**Fig. 5:**
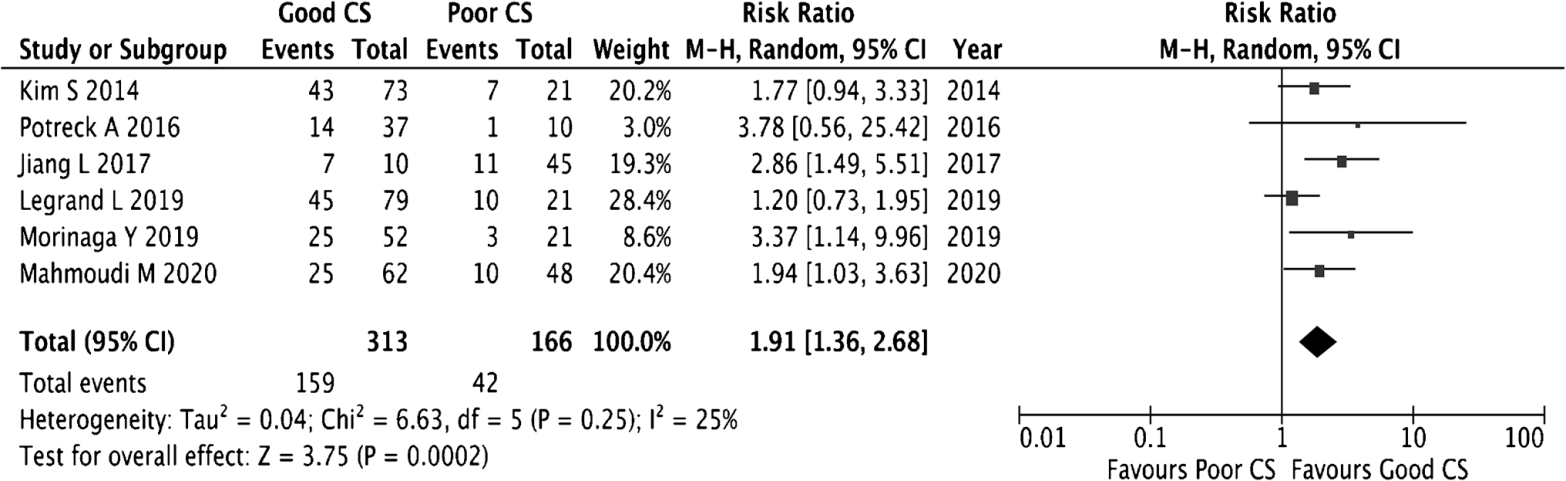
Forest plot displaying the overall risk-ratios of pre-treatment CS (good vs poor) and favorable outcome mRS (0–2) at 90 days in thrombectomy treated patients using a random effects model. CS, collateral status; CI, confidence interval

Of 25 included studies, 6 (30%) qualified for the quantitative synthesis for correlating CS with functional independence. All 6 studies were retrospective in design with a total of 479 patients treated with thrombectomy, 313 patients (50.8%) with good CS and 166 with poor CS (25.3%). In Fig. 5, the forest plot shows that good collateral status (defined in a dichotomy vs poor collaterals for 4 studies and vs no collaterals in 2 studies, see Supplement Table 4) identified through the various MRI techniques significantly correlated with good functional outcome (mRS=0–2, i.e., independence) (RR 1.91, 95%CI [1.36, 2.68], *p*= 0.0002); that is almost double the rate of functional independence with overlapping effect estimates between studies and no sign of statistical heterogeneity (*I*^2^=25%). Visual inspection of the funnel plot shows asymmetry with lack of studies in the lower left-hand side indicating potential publication bias, supplemental figure 1.

### Sensitivity analyses

**Fig. 6:**
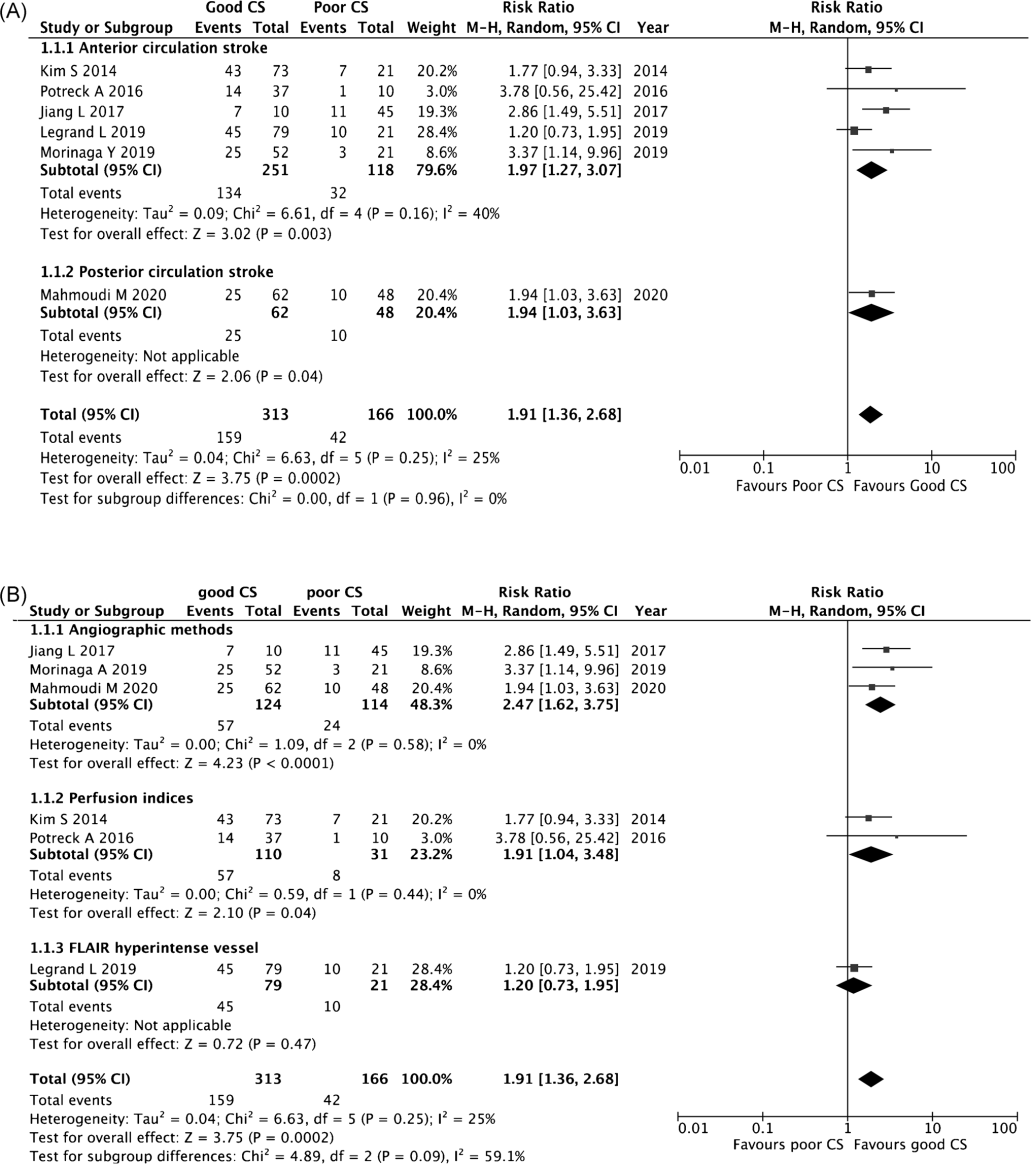
Sensitivity analyses for functional independence (mRS=0-–2) at 90 days for (**A**) affected arterial territory; (**B**) MR methods

Results of differences between subgroups divided by the MRI method and affected arterial territory can be seen in Fig. 6. Comparing arterial territory subgroups there is no statistically significant difference in rate of functional independence between anterior and posterior circulation stroke (*p*=0.96) with RR of 1.97 and 1.94 respectively. Between the different MR methods we assessed, good collaterals evaluated by angiographic methods and perfusion indices are independently associated with better outcome, RR 2.47 and 1.91 respectively. However, there was substantial heterogeneity between MR method subgroups (*I*^2^=59%).

## Discussion

In this systematic review and meta-analysis, we demonstrate that in stroke patients with LVO, good pre-thrombectomy collaterals assessed using MRI were associated with higher rates of functional independence at 90 days compared to patients with poor collaterals. Our study reviewed a range of MRI methods by including direct and indirect routes of assessment unlike a previous review that excluded indirect methods [4]. This allowed us to explore the different independent predictors of CS and the cut-offs used for dichotomizing CS into good and poor. Most of the MRI methods we identified utilized indirect surrogates for CS assessment such as derivation from perfusion indices, FHVs, and impact of cerebral small vessel disease. Even including direct and indirect methods combined, CS was assessed for 2467 patients pre-thrombectomy, which is relatively small compared to 5058 and 3542 in previous systematic reviews of CT and DSA in thrombectomy, respectively [6, 7]. Nevertheless, our numbers are compatible with the meta-analysis of individual patient-level data from 7 thrombectomy RCTs where 1388 patients underwent CT compared to only 364 patients for MRI [2].

The commonest MRI methodology we identified was derived from perfusion techniques. This is perhaps not surprising given that thrombectomy trials that included MRI tended to enroll patients based on the diffusion-perfusion mismatch concept (small lesion core according to diffusion versus larger salvageable penumbra on perfusion, Fig. 4) rather than evaluating collaterals per se. Examples are the SWIFT-PRIME, DEFUSE3, and DAWN [13, 14, 48] trials that enrolled patients with imaging evidence of salvageable brain tissue (albeit at extended time periods in the latter two trials) resulting in higher rates of good functional outcome compared to studies that did not use advanced imaging including the MR-CLEAN, REVASCAT, THRACE, and PISTE trials [9–11, 15]. These methods mainly detect parenchymal perfusion which is probably indirect evidence of microcirculation that sustains ischemic penumbra, i.e., temporary enhancement in microvascular perfusion that persists after the time of insult and is thought to be the effect of collaterals [16]. In the DEFUSE2 cohort, HIR was used as an independent predictor of final infarct volume and results showed that low HIR is associated with slower infarct growth and functional independence at 90 days [49]. Results were similar to other studies in our review that utilized HIR as an indicator of CS supporting the hypothesis that HIR provides a good estimate of collateral status. While the proportions of time-to-maximum (Tmax) lesions varied, all these studies employed a similar cut-off (0.35–0.4) which adds a degree of generalizability to this method. However, deriving CS from MR perfusion indices requires dedicated post-processing software that is rarely available in clinical practice and not adequately validated due to the small samples and non-unified cut-offs used in testing [50]. Additionally, since it is highly convenient to retrieve data from workstations for further post-processing at any time point, 90% of the studies were retrospective in design which automatically introduces a risk of bias. Even when retrospective studies reflect actual clinical practice, they might overlook eligible patients and miss data points [51]. This necessitates prospective evaluation to overcome the limitations of small samples and over exclusions.

Another indicator of collateral status that was reported among some studies in our review is the presence of hyperintense vessels on FLAIR (Fig. 3). FHVs are thought to result from abnormal blood flow in the collaterals distal to the site of occlusion. Normally, on FLAIR, vessels appear dark due to lack of returned signal from energized blood that has moved out of the vessel (and thus out of the imaging field of view). However, when flow is altered due to steno-occlusive disease, signal can be detected within these vessels and this may represent leptomeningeal collaterals with retrograde and perhaps sluggish flow sustaining salvageable tissue [52]. Previous studies have shown that FHVs predict vessel occlusion and were more commonly associated with MCA territory strokes [53]. We similarly observed in our review that FVHs were present in patients with LVO in the MCA territory and were associated with good collateral status. However, among the imaging analysis methods we reviewed, the grading of collaterals and proposed cut-offs varied widely.

While MRA sequences in stroke protocols are important for the assessment of occlusion location and clot length [54, 55], in our review, direct angiographic methods for collateral assessment were few compared to the perfusion methods described above. CE-MRA and TOF-MRA sequences were used with and without contrast (Fig. 2), whereas a good collateral circulation on MRA had various definitions including (a) presence and patency of the primary collateral vessel of interest, (b) sufficient number of collaterals detected on the occluded side compared with the patent side, and (c) complete leptomeningeal filling on dynamic MRA. Reporting of angiographic images in clinical practice is usually qualitative and does not require sophisticated post-processing software. In a previous systematic review exploring the reliability of assessing CS, one study that utilized MRA was assessed and showed near-perfect interobserver agreement (Kappa 0.93) [5]. It is unclear why published data are less available for methods that are simpler and less time consuming to acquire and to assess. Possibly, studies that evaluated perfusion methods, even when MRA images were acquired as part of the same protocol, simply did not assess the MRA data in this context. Unlike perfusion sequences, MRA assesses the primary and secondary collateral vasculature and occlusion location simultaneously while minimizing scan times. It is critical to focus on enhancing the quality of studies that incorporate simple techniques because they are easier to translate to clinical practice. Since angiographic sequences are already implemented in routine stroke protocols worldwide for occlusion location, evaluating CS pre-thrombectomy should not be difficult on a larger scale.

To the best of our understanding, our review is the first to correlate CS with functional independence using MRI; however, unlike a review assessing CTA and DSA, we had fewer studies available for meta-analysis, so subgroups were few and underpowered. We were limited by the presentation of data for the numbers of patients with good and poor collaterals (e.g., as mean or median) while in other studies, mRS at 90 days was not specified for each group of CS. Secondly, standard grading systems have been developed specifically for the reporting of CT and DSA images [56], making it relatively easy to consistently report CS in these studies, whereas no commonly used grading methods have been designed for the more novel MRI techniques. Thus, CS was rarely explicitly specified as good or poor on MRI. Also, the dichotomization method used to group collaterals might have inflated the number of participants with good CS by joining good and moderate into one category. We combined patients within and beyond 6 h of stroke onset, but the impact of collateral status on functional outcome after thrombectomy may differ between these groups. It is possible that the sequence order in a given stroke MRI protocol might affect the extent of visible collaterals if time of flight MRA follows contrast-enhanced MR perfusion (potentially increasing visible collaterals in the presence of contrast). We were not able to assess or control for this. Lastly, our review only correlates with functional independence as the outcome of efficacy and did not study the association with symptomatic intracranial hemorrhage (sICH) or mortality.

With a drive to deliver more patients efficiently for thrombectomy, and with various MR imaging methods for collateral scoring emerging, translating potential improvements to clinical practice requires care because (a) clinical imaging departments need to establish criteria on how to incorporate collateral status in brain MRI radiology reports for prognostication purposes [57]; (b) only a few of the thrombectomy RCTs explicitly incorporated collateral assessment in their criteria and most post hoc analyses suffer from small sample sizes. So even with compelling evidence that patients with preserved collaterals display higher rates of functional independence after thrombectomy, such results should be interpreted with caution. Most trials did not include patients with poor or malignant collaterals; thus, we have no evidence from which to plan for these patients and there is a risk that those with less favorable imaging are inappropriately excluded from accessing a highly effective therapy; (c) MRI is not widely routinely used for baseline stroke assessment worldwide and it is unclear whether greater implementation is worthwhile for individual stroke units. Rather, we hoped to improve understanding of MRI collateral assessment for centers that already routinely use MRI. Future studies should focus on (a) incorporating MRI in prospective studies, (b) standardizing a collateral grading system for MRI; and (c) performing studies of diagnostic test accuracy to explore the best MRI technique for evaluating CS.

In conclusion, our study shows that the presence and high quality of cerebral collaterals nearly doubled the rate of good outcome and is a promising predictor of functional independence in acute ischemic stroke patients prior to thrombectomy. While MRI methods are continuously evolving, there is inconsistency in techniques and grading methods due to MRI being understudied and commonly seen as being unfeasible in the acute setting. If, as several thrombectomy RCTs seem to suggest, that MRI collateral assessment could become an integral part of pre-thrombectomy assessment especially in centers where this can be routinely delivered, the methods need to be validated, especially simple qualitative approaches for their wide applicability.

## Supplementary information

Supplementary materials 1: Supplemental Table 1. PRISMA checklist. Supplemental Table 2. Search strategy. Supplemental Table 3. Data extraction form. Supplemental Table 4. Dichotomization process of selected studies for the metanalysis. Supplemental Table 5. Quality assessment of studies for meta-analysis. Supplemental Figure 1: Funnel plot showing asymmetry (uneven distribution) with loss of studies in the lower left-hand side of the plot.
