# Supplementary material for: MRI for collateral assessment pre-thrombectomy and association with outcome: a systematic review and meta-analysis
Source: Neuroradiology. 2023 Feb 27;65(6):1001–14. doi: 10.1007/s00234-023-03127-8 (PMC10169893; doi:10.1007/s00234-023-03127-8)
Supplement: Supplementary file 1 — Supplemental Table 1. PRISMA checklist. Supplemental Table 2. Search strategy. Supplemental Table 3. Data extraction form. Supplemental Table 4. Dichotomization process of selected studies for the metanalysis. Supplemental Table 5. Quality assessment of studies for meta-analysis. Supplemental Figure 1: Funnel plot showing asymmetry (uneven distribution) with loss of studies in the lower left-hand side of the plot. [file 234_2023_3127_MOESM1_ESM.docx]

*MRI for collateral assessment pre-thrombectomy and association with outcome: A systematic review and meta-analysis*

## SUPPLEMENTAL MATERIAL

Sarah Emhemed Abousrafa (MSc)^1^, Dr Grant Mair (MD)^2^.

1. College of medicine and veterinary medicine, University of Edinburgh, UK.

2. Centre for Clinical Brain Sciences, University of Edinburgh, UK.

**Supplemental Table 1.** PRISMA checklist

| **Section and Topic** | **Item #** | **Checklist item** | **Location where item is reported** |
| --- | --- | --- | --- |
| **TITLE** | | |  |
| Title | 1 | Identify the report as a systematic review. | Page 1 |
| **ABSTRACT** | | |  |
| Abstract | 2 | See the PRISMA 2020 for Abstracts checklist. | Page 1 |
| **INTRODUCTION** | | |  |
| Rationale | 3 | Describe the rationale for the review in the context of existing knowledge. | Page 1,2 |
| Objectives | 4 | Provide an explicit statement of the objective(s) or question(s) the review addresses. |  |
| **METHODS** | | |  |
| Eligibility criteria | 5 | Specify the inclusion and exclusion criteria for the review and how studies were grouped for the syntheses. | Page 4 |
| Information sources | 6 | Specify all databases, registers, websites, organisations, reference lists and other sources searched or consulted to identify studies. Specify the date when each source was last searched or consulted. | Page 4 |
| Search strategy | 7 | Present the full search strategies for all databases, registers and websites, including any filters and limits used. | Page 5 |
| Selection process | 8 | Specify the methods used to decide whether a study met the inclusion criteria of the review, including how many reviewers screened each record and each report retrieved, whether they worked independently, and if applicable, details of automation tools used in the process. | Page 5 |
| Data collection process | 9 | Specify the methods used to collect data from reports, including how many reviewers collected data from each report, whether they worked independently, any processes for obtaining or confirming data from study investigators, and if applicable, details of automation tools used in the process. |  |
| Data items | 10a | List and define all outcomes for which data were sought. Specify whether all results that were compatible with each outcome domain in each study were sought (e.g. for all measures, time points, analyses), and if not, the methods used to decide which results to collect. | Page 6 |
|  | 10b | List and define all other variables for which data were sought (e.g. participant and intervention characteristics, funding sources). Describe any assumptions made about any missing or unclear information. |  |
| Study risk of bias assessment | 11 | Specify the methods used to assess risk of bias in the included studies, including details of the tool(s) used, how many reviewers assessed each study and whether they worked independently, and if applicable, details of automation tools used in the process. | Page 6 |
| Effect measures | 12 | Specify for each outcome the effect measure(s) (e.g. risk ratio, mean difference) used in the synthesis or presentation of results. | Page 7 |
| Synthesis methods | 13a | Describe the processes used to decide which studies were eligible for each synthesis (e.g. tabulating the study intervention characteristics and comparing against the planned groups for each synthesis (item #5)). | Page 6 |
|  | 13b | Describe any methods required to prepare the data for presentation or synthesis, such as handling of missing summary statistics, or data conversions. | NA |
|  | 13c | Describe any methods used to tabulate or visually display results of individual studies and syntheses. | Page 7  Page 7 |
|  | 13d | Describe any methods used to synthesize results and provide a rationale for the choice(s). If meta-analysis was performed, describe the model(s), method(s) to identify the presence and extent of statistical heterogeneity, and software package(s) used. |  |
|  | 13e | Describe any methods used to explore possible causes of heterogeneity among study results (e.g. subgroup analysis, meta-regression). |  |
|  | 13f | Describe any sensitivity analyses conducted to assess robustness of the synthesized results. |  |
| Reporting bias assessment | 14 | Describe any methods used to assess risk of bias due to missing results in a synthesis (arising from reporting biases). | NA |
| Certainty assessment | 15 | Describe any methods used to assess certainty (or confidence) in the body of evidence for an outcome. |  |
| **RESULTS** | | |  |
| Study selection | 16a | Describe the results of the search and selection process, from the number of records identified in the search to the number of studies included in the review, ideally using a flow diagram. | Page 7  Figure 1 |
|  | 16b | Cite studies that might appear to meet the inclusion criteria, but which were excluded, and explain why they were excluded. | Page 7 |
| Study characteristics | 17 | Cite each included study and present its characteristics. | Page 9  Table 1 |
| Risk of bias in studies | 18 | Present assessments of risk of bias for each included study. | Page 9 |
| Results of individual studies | 19 | For all outcomes, present, for each study: (a) summary statistics for each group (where appropriate) and (b) an effect estimate and its precision (e.g. confidence/credible interval), ideally using structured tables or plots. | Figure 2 |
| Results of syntheses | 20a | For each synthesis, briefly summarise the characteristics and risk of bias among contributing studies. | Page 9,16  Figure 3 |
|  | 20b | Present results of all statistical syntheses conducted. If meta-analysis was done, present for each the summary estimate and its precision (e.g. confidence/credible interval) and measures of statistical heterogeneity. If comparing groups, describe the direction of the effect. |  |
|  | 20c | Present results of all investigations of possible causes of heterogeneity among study results. |  |
|  | 20d | Present results of all sensitivity analyses conducted to assess the robustness of the synthesized results. |  |
| Reporting biases | 21 | Present assessments of risk of bias due to missing results (arising from reporting biases) for each synthesis assessed. | NA |
| Certainty of evidence | 22 | Present assessments of certainty (or confidence) in the body of evidence for each outcome assessed. |  |
| **DISCUSSION** | | |  |
| Discussion | 23a | Provide a general interpretation of the results in the context of other evidence. | Page 19 |
|  | 23b | Discuss any limitations of the evidence included in the review. |  |
|  | 23c | Discuss any limitations of the review processes used. |  |
|  | 23d | Discuss implications of the results for practice, policy, and future research. |  |

*From:*  Page MJ, McKenzie JE, Bossuyt PM, et al. The PRISMA 2020 statement: an updated guideline for reporting systematic reviews. BMJ 2021;372:n71. doi:10.1136/bmj.n

NA; not available.

## Supplemental Table 2. Search strategy

| **MEDLINE search** |
| --- |

1 exp Stroke/

2 exp Cerebrum/ and exp infarction/

3 exp Cerebral Arteries/ and exp arterial occlusive disease/

4 exp Brain Ischemia/

5 exp Stroke/ and exp arterial occlusive disease/

6 exp Brain Ischemia/dg [Diagnostic Imaging]

7 exp Arterial Occlusive Diseases/pa [Pathology]

8 exp Arterial Occlusive Diseases/

9 exp Intracranial Embolism/

10 exp Stroke, Lacunar/

11 exp Intracranial Thrombosis/

12 exp Brain Infarction/

13 exp Cerebral Infarction/

14 exp Cerebrovascular Disorders/

15 exp Cerebral Arteries/ and exp thrombus/

16 stroke.tw.

17 acute stroke.tw.

18 acute ischemic stroke.tw.

19 acute ischaemic stroke.tw.

20 cerebral stroke.tw.

21 cerebrovascular accident.tw.

22 CVA.tw.

23 (acute adj1 ischemic adj1 stroke).tw.

24 (acute adj1 stroke).tw.

25 anterior circulation stroke.tw.

26 brain stroke.tw.

27 cerebral isch$.tw.

28 intracranial vessel occlusion.tw.

29 intracranial artery occlusion.tw.

30. intracranial thromb$.tw.

31 cerebral artery occlusion.tw.

32 large artery occlusion.tw.

33 (cerebral adj isch$ adj injury).tw.

34 AIS.tw.

35 middle cerebral artery occlusion.tw.

36 MCA occlusion.tw.

37 stroke.ti.

38 (stroke adj thrombectomy).tw.

39 exp Collateral Circulation/

40 exp Collateral Circulation/ph [Physiology]

41 collateral circulation.tw.

42 brain collateral$.tw.

43 leptomeningeal collateral.tw.

44 collateral blood vessels.tw.

45 collateral vessels.tw.

46 collateral flow.tw.

47 collateral blood supply.tw.

48 collateral supply.tw.

49 (collateral adj1 circulation).tw.

50 collateral map.tw.

51 collateral status.tw.

52 exp Collateral Circulation/dg [Diagnostic Imaging]

53 collateral filling.tw.

54 collateral scoring.tw.

55 collateral grading.tw.

56 (collateral adj system).tw.

57 ASITN SIR.tw.

58 collateral scale.tw.

59 collateral pathways.tw.

60 exp Magnetic Resonance Imaging/

61 exp Magnetic Resonance Angiography/

62 exp Magnetic Resonance Imaging/mt [Methods]

63 exp Diffusion Magnetic Resonance Imaging/

64 exp magnetic resonance imaging/ and exp Cerebrum/

65 magnetic resonance imaging/ and Multimodal Imaging/

66 exp magnetic resonance imaging/ and exp Stroke/

67 exp magnetic resonance imaging/ and exp cerebrovascular circulation/

68 exp magnetic resonance imaging/ and collateral circulation/

69 exp magnetic resonance imaging/ and exp Cerebral Angiography/

70 magnetic resonance imaging.tw.

71 magnetic resonance angiography.tw.

72 MRI.tw.

73 MR angiog$.tw.

74 (magnetic adj1 resonance adj1 imaging).tw.

75 (TOF adj1 MRA).tw.

76 time-of-flight.tw.

77 MR Perfusion.tw.

78 MRI perfusion.tw.

79 3D MRI.tw.

80 three-dimensional MRI.tw.

81 4D MRA.tw.

82 exp Thrombectomy/

83 exp Thrombectomy/mt [Methods]

84 exp Stroke/su [Surgery]

85 exp Vascular Surgical Procedures/

86 exp Brain Ischemia/th [Therapy]

87 exp Thrombectomy/ and exp Stroke/

88 exp Arterial Occlusive Diseases/th [Therapy]

89 exp Stroke/th [Therapy]

90 exp Mechanical Thrombolysis/

91 exp Thrombectomy/ and exp Arterial Occlusive Diseases/

92 thrombectomy.tw.

93 endovascular thrombectomy.tw.

94 recanalisation.tw.

95 recanalization.tw.

96 (thrombectomy adj reperfusion).tw.

97 (thrombectomy adj outcome).tw.

98 mechanical thrombectomy.tw.

99 cerebral artery thrombectomy.tw.

100 thrombectomy-treated.tw.

101 (endovascular adj1 thrombectomy).tw.

102 endovascular treatment.tw.

103 EVT.tw.

104 endovascular procedure$.tw.

105 exp Diagnostic Imaging/

106 exp "Predictive Value of Tests"/

107 "Reproducibility of Results"/

108 exp Prognosis/

109 exp "Sensitivity and Specificity"/

110 exp Randomized Controlled Trial/

111 sensitivity.tw.

112 specificity.tw.

113 (sensitivity adj specificity).tw.

114 positive predictive value.tw.

115 negative predictive value.tw.

116 false positive.tw.

117 false negative.tw.

118 true negative.tw.

119 true positive.tw.

120 likelihood ratio$.tw.

121 accuracy.tw.

122 test accuracy.tw.

123 randomized controlled trials.tw.

124 randomised controlled trials.tw.

125 RCT.tw.

126 1 or 2 or 3 or 4 or 5 or 6 or 7 or 8 or 9 or 10 or 11 or 12 or 13 or 14 or 15 or 16 or 17 or 18 or 19 or 20 or 21 or 22 or 23 or 24 or 25 or 26 or 27 or 28 or 29 or 30 or 31 or 32 or 33 or 34 or 35 or 36 or 37 or 38

127 39 or 40 or 41 or 42 or 43 or 44 or 45 or 46 or 47 or 48 or 49 or 50 or 51 or 52 or 53 or 54 or 55 or 56 or 57 or 58 or 59

128 60 or 61 or 62 or 63 or 64 or 65 or 66 or 67 or 68 or 69 or 70 or 71 or 72 or 73 or 74 or 75 or 76 or 77 or 78 or 79 or 80 or 81

129 82 or 83 or 84 or 85 or 86 or 87 or 88 or 89 or 90 or 91 or 92 or 93 or 94 or 95 or 96 or 97 or 98 or 99 or 100 or 101 or 102 or 103 or 104

130 105 or 106 or 107 or 108 or 109 or 110 or 111 or 112 or 113 or 114 or 115 or 116 or 117 or 118 or 119 or 120 or 121 or 122 or 123 or 124 or 125

131 126 and 127 and 128 and 129 and 130

132 limit 131 to "review articles"

133 limit 131 to animals

134 131 and "Case Reports" [Publication Type]

135 131 and "Comment" [Publication Type]

| **EMBASE search** |
| --- |

1 exp cerebrovascular accident/

2 exp brain ischemia/

3 exp peripheral occlusive artery disease/

4 exp cerebrovascular accident/ and exp artery occlusion/

5 exp brain infarction/

6 exp brain embolism/

7 exp lacunar stroke/

8 exp occlusive cerebrovascular disease/

9 exp brain artery/ and exp thrombus/

10 stroke.tw.

11 acute stroke.tw.

12 acute ischaemic stroke.tw.

13 acute ischemic stroke.tw.

14 cerebral stroke.tw.

15 cerebrovascular accident.tw.

16 CVA.tw.

17 anterior circulation stroke.tw.

18 cerebral isch$.tw.

19 intracranial vessel occlusion.tw.

20 intracranial artery occlusion.tw.

21 intracranial thromb$.tw.

22 large artery occlusion.tw.

23 AIS.tw.

24 middle cerebral artery occlusion.tw.

25 MCA occlusion.tw.

26 stroke.ti.

27 (stroke adj thrombectomy).tw.

28 exp collateral circulation/

29 collateral circulation.tw.

30 brain collateral$.tw.

31 leptomeningeal collateral.tw.

32 collateral blood vessels.tw.

33 collateral vessels.tw.

34 collateral flow.tw.

35 collateral blood supply.tw.

36 collateral supply.tw.

37 (collateral adj1 circulation).tw.

38 collateral map.tw.

39 collateral status.tw.

40 collateral filling.tw.

41 collateral scoring.tw.

42 collateral grading.tw.

43 ASITN SIR.tw.

44 collateral scale.tw.

45 collateral pathways.tw.

46 exp nuclear magnetic resonance imaging/

47 exp magnetic resonance angiography/

48 exp diffusion weighted imaging/

49 magnetic resonance imaging.tw.

50 magnetic resonance angiography.tw.

51 MRI.tw.

52 MR angio$.tw.

53 (magnetic adj1 resonance adj1 imaging).tw.

54 (TOF adj MRA).tw.

55 time-of-flight.tw.

56 MR perfusion.tw.

57 MRI perfusion.tw.

58 3D MRI.tw.

59 three-dimensional MRI.tw.

60 4D MRA.tw.

61 QMRA.tw.

62 exp diagnostic imaging/

63 exp predictive value/

64 exp reproducibility/

65 exp prognosis/

66 exp "sensitivity and specificity"/

67 exp "randomized controlled trial (topic)"/ or exp "controlled clinical trial (topic)"/

68 sensitivity.tw.

69 specificity.tw.

70 (sensitivity adj specificity).tw.

71 positive predictive value.tw.

72 negative predictive value.tw.

73 false positive.tw.

74 false negative.tw.

75 true negative.tw.

76 true positive.tw.

77 likelihood ratio$.tw.

78 accuracy.tw.

79 test accuracy.tw.

80 randomized controlled trials.tw.

81 randomised controlled trials.tw.

82 diagnostic accuracy.tw.

83 exp thrombectomy/

84 exp mechanical thrombectomy/

85 exp vascular surgery/

86 exp brain ischemia/th [Therapy]

87 exp thrombectomy/ and exp cerebrovascular accident/

88 exp artery occlusion/th [Therapy]

89 thrombectomy.tw.

90 mechanical thrombectomy.tw.

91 cerebral artery thrombectomy.tw.

92 endovascular thrombectomy.tw.

93 recanalisation.tw.

94 recanalization.tw.

95 (thrombectomy adj reperfusion).tw.

96 (thrombectomy adj outcome).tw.

97 thrombectomy-treated.tw.

98 (endovascular adj1 thrombectomy).tw.

99 endovascular treatment.tw.

100 EVT.tw.

101 endovascular procedu$.tw.

102 1 or 2 or 3 or 4 or 5 or 6 or 7 or 8 or 9 or 10 or 11 or 12 or 13 or 14 or 15 or 16 or 17 or 18 or 19 or 20 or 21 or 22 or 23 or 24 or 25 or 26 or 27

103 28 or 29 or 30 or 31 or 32 or 33 or 34 or 35 or 36 or 37 or 38 or 39 or 40 or 41 or 42 or 43 or 44 or 45

104 46 or 47 or 48 or 49 or 50 or 51 or 52 or 53 or 54 or 55 or 56 or 57 or 58 or 59 or 60 or 61

105 62 or 63 or 64 or 65 or 66 or 67 or 68 or 69 or 70 or 71 or 72 or 73 or 74 or 75 or 76 or 77 or 78 or 79 or 80 or 81 or 82

106 83 or 84 or 85 or 86 or 87 or 88 or 89 or 90 or 91 or 92 or 93 or 94 or 95 or 96 or 97 or 98 or 99 or 100 or 101

107 102 and 103 and 104 and 105 and 106

108 limit 107 to animals

109 107 and "Review" [Publication Type]

110 107 and "Conference Abstract" [Publication Type]

## Supplemental Table 3. Data extraction form

| ***# of study on database list ( )*** | | | | | | | | | | ***Location*** |
| --- | --- | --- | --- | --- | --- | --- | --- | --- | --- | --- |
| *Author* |  | | | | | | | | |  |
| *Year of publication* |  | | | | | | | | |  |
| *Study title* |  | | | | | | | | |  |
| *Database* |  | | | | | | | | |  |
| *Publication type* |  | | | | | | | | |  |
| ***Minimal eligibility ( For title and abstract screening )*** | | | | | | | | | | |
| *Assessed CS pre-thrombectomy with any MRI method* | Yes | | | No | | | Not sure | | |  |
|  |  | | |  | | |  | | |  |
| ***General Study characteristics*** | | | | | | | | | | |
| *Study design* | Prospective | | | | Retrospective | | | | |  |
|  |  | | | |  | | | | |  |
| *Sample size* | n. patients treated with EVT | | | |  | | | | |  |
|  | n. patients treated with tPA | | | |  | | | | |  |
| *Onset time* | Mentioned | | Not mentioned | | Not sure | | | Time in hours | |  |
|  |  | |  | |  | | |  | |  |
| *Assessed CS with the reference standard DSA* | Yes | | | | No | | | | |  |
|  |  | | | |  | | | | |  |
| Affected arterial territory |  | | | | | | | | |  |
| ***MRI method*** | | | | | | | | | | |
| *How was CS evaluated ?* |  | | | | | | | | |  |
| *Magnetic field strength* | *1.5 T* | | *3T* | | | *Both* | | | *Not mentioned* |  |
|  |  | |  | | |  | | |  |  |
| *Grading method* |  | | | | | | | | |  |
| *Cut-off* | *Value of Cut-off* | | | | | *Method of determining cut-off* | | | |  |
|  |  | | | | |  | | | |  |
| ***Outcome*** | | | | | | | | | | |
| *Favorable outcome*  *( Modified Rankin score )* | *Yes* | *No* | *Duration of outcome* | | | *Specified mRS range* | | | *Missing data* |  |
|  |  |  |  | | |  | | |  |  |
| *Results* | *Intervention* | | | | | *Comparison* | | | |  |
|  | *Events* | | *Total* | | | *events* | | | *total* |  |
|  | No. with Good CS  ( ) | | No. with good CS + favorable outcome  ( ) | | | No. with poor CS  ( ) | | | No. with poor CS + favorable outcome  ( ) |  |
| *Notes :* |  | | | | | | | | |  |

**Supplemental Table 4.** Dichotomization process of selected studies for the metanalysis

| *Author* | *Collateral score* | | |
| --- | --- | --- | --- |
| *Mahmoudi M et al.* ^28^ | Posterior circulation collateral score (0-2) | | |
|  | Trichotomy | Dichotomy | |
|  | 0=no PCOM  1=Unilateral  2=Bilateral | ( good collaterals )  (1-2)  Unilateral  bilateral | ( poor collaterals )  (0)  None |
| *Kim SJ et al.* ^20^ | MRP Based collateral grade (ASITN/SIR) | | |
|  | Trichotomy | Dichotomy | |
|  | Poor (grade 1)  Intermediate/good (grade 2-3)  excellent (grade 4) | ( good collaterals )  (grade 2-4) | ( poor collaterals )  (grade 1) |
| *Legrand L et al.* ^23^ | FVH-DWI mismatch rated as present or absent | | |
|  | - | Dichotomy | |
|  |  | ( good collaterals )  with FVH-DWI mismatch | ( poor collaterals )  No FVH-DWI  mismatch |
| *Morinaga Y et al.* ^33^ | Presence/absence of ACOMA | | |
|  | - | Dichotomy | |
|  |  | ( good collaterals)  Present A-Com | ( poor collaterals )  No A-Com |
| *Potreck A et al.* ^40^ | TMACS correlated with ASITN/SIR | | |
|  | Trichotomy | Dichotomy | |
|  | Good (grade 4)  Moderate (grade 3)  Poor (grade 1-2) | ( good collaterals )  Grade (3-4) | ( poor collaterals )  Grade (1-2) |
| *Jiang L et al.* ^39^ | Collaterals of the affected side compared with the patent side Grades 1-5 | | |
|  |  | Dichotomy | |
|  | 5 point scale  (1) Absent, (2) less than patent side, (3)equal to patent side, (4)greater than patent side, (5)exuberant. | ( good collaterals )  (grade ≥ 3) | ( poor collaterals )  (grade≤2) |

*PCOM; posterior communicating artery, MRP; magnetic resonance perfusion, ASITN/SIR; American Society of Interventional and Therapeutic Neuroradiology/Society of Interventional Radiology scale, FVH-DWI; FLAIR vascular hyperintensity-diffusion weighted imaging, A-Com; anterior communicating, TMACS; Tmax map-assessed collateral score.*

**Supplemental Table 5.** Quality assessment of studies for meta-analysis

|  | | Mahmoudi M et al.^28^ | Morinaga Y. et al.^33^ | Legrand L  et al.^23^ | Jiang L  et al.^39^ | Potreck A  et al.^40^ | Kim SJ  et al.^20^ |
| --- | --- | --- | --- | --- | --- | --- | --- |
| **Selection** | *Representativeness of exposed cohort* | * | * | * | * | 0 | * |
|  | *Selection of non-exposed cohort* | 0 | 0 | 0 | 0 | 0 | * |
|  | *Ascertainment of exposure* | * | * | * | * | * | * |
|  | *Outcome of interest was not present at start of the study* | 0 | * | * | * | 0 | * |
| **Comparability**  **items** | *Age* | 0 | * | 0 | * | * | * |
|  | *NIHSS, sex, comorbidities* | * | * | * | * | * | * |
| **Outcome** | *Assessment of outcome of interest* | * | * | * | * | * | * |
|  | *follow-up was long enough for outcome to occur* | * | 0 | * | * | * | * |
|  | *Adequacy of follow-up* | * | * | * | 0 | 0 | * |
|  | *Total* | 6 | 7 | 7 | 7 | 5 | 9 |

(*)Fulfilled criteria

(0) Did not fulfil criteria

**Supplemental Figure 1:** Funnel plot showing asymmetry (uneven distribution) with loss of studies in the lower left-hand side of the plot.
